# Circadian Rhythms in Legumes: What Do We Know and What Else Should We Explore?

**DOI:** 10.3390/ijms22094588

**Published:** 2021-04-27

**Authors:** Hazel Marie Kugan, Nur Ardiyana Rejab, Nurul Amylia Sahruzaini, Jennifer Ann Harikrishna, Niranjan Baisakh, Acga Cheng

**Affiliations:** 1 Faculty of Science, Institute of Biological Sciences, Universiti Malaya, Kuala Lumpur 50603, Malaysia; s2005438@siswa.um.edu.my (H.M.K.); ardiyana@um.edu.my (N.A.R.); nurulamylia@um.edu.my (N.A.S.); jennihari@um.edu.my (J.A.H.); 2 Centre for Research in Biotechnology for Agriculture (CEBAR), Universiti Malaya, Kuala Lumpur 50603, Malaysia; 3 School of Plant, Environmental, and Soil Science, Louisiana State University Agricultural Center, Baton Rouge, LA 70803, USA; NBaisakh@agcenter.lsu.edu

**Keywords:** circadian rhythmicity, climate change, crop development, legumes, plant molecular biology

## Abstract

The natural timing devices of organisms, commonly known as biological clocks, are composed of specific complex folding molecules that interact to regulate the circadian rhythms. Circadian rhythms, the changes or processes that follow a 24-h light–dark cycle, while endogenously programmed, are also influenced by environmental factors, especially in sessile organisms such as plants, which can impact ecosystems and crop productivity. Current knowledge of plant clocks emanates primarily from research on Arabidopsis, which identified the main components of the circadian gene regulation network. Nonetheless, there remain critical knowledge gaps related to the molecular components of circadian rhythms in important crop groups, including the nitrogen-fixing legumes. Additionally, little is known about the synergies and trade-offs between environmental factors and circadian rhythm regulation, especially how these interactions fine-tune the physiological adaptations of the current and future crops in a rapidly changing world. This review highlights what is known so far about the circadian rhythms in legumes, which include major as well as potential future pulse crops that are packed with nutrients, particularly protein. Based on existing literature, this review also identifies the knowledge gaps that should be addressed to build a sustainable food future with the reputed “poor man’s meat”.

## 1. Introduction

Circadian rhythms are broadly defined as endogenous biological processes that occur within an oscillation of approximately 24 h. Also referred to as biological clocks, circadian rhythms are subject to both environmental entrainment and temperature compensation [1]. This phenomenon enables organisms to anticipate periodic changes in the environment and subsequently adjust or synchronize their developmental and physiological responses to the best time of the day and make efficient use of available resources [2]. Efficient resource management is especially important for sessile organisms, such as plants, which cannot evade unfavorable conditions [2]. In general, the circadian rhythms in plants, which are often regulated by multiple feedback loops, have a higher complexity than those in animals, which are governed mostly by a centralized pacemaker [1,3]. Plants have multiple tissue- and organ-specific clocks that allow fine-tuning of physiological adaptations to changing environmental conditions. Plant circadian rhythms are key to plant survival in diverse environmental conditions [4] and hence, have become a major focus in plant research. Understanding plant circadian rhythm and its manipulation may revolutionize modern food crop production and enhance food security [3,5].

**Figure 1:**
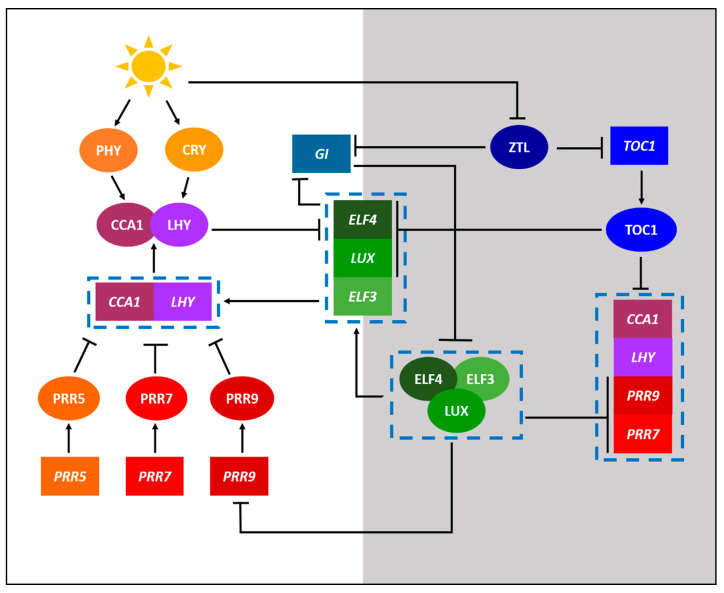
A simplified model displaying the core components of the circadian gene regulation network of model plant *Arabidopsis thaliana.* Components with a white and grey background depict day and night processes, respectively. The circadian regulation of *A. thaliana* consists of a complex interconnected series of feedback loops. Activation is indicated by lines with arrowheads while repression is indicated by lines with perpendicular heads. Proteins are indicated by ellipses while genes are indicated by rectangles. Genes and proteins acting concurrently or as a complex are indicated within the dashed boxes.

The current state of knowledge of biological clocks in plants stems primarily from the research on the model plant species Arabidopsis (*Arabidopsis thaliana*) through a combination of omics approaches [1]. Figure 1 shows a simplified circadian gene regulation network of Arabidopsis, illustrating various physiological processes that are influenced by circadian rhythmicity. The core loop, which is interconnected with the morning and evening loops, consists of the main Myb-related transcription factors; CIRCADIAN CLOCK ASSOCIATED 1 (CCA1) and LATE ELONGATED HYPOCOTYL (LHY), and the transcriptional repressor TIMING OF CAB EXPRESSION 1 (TOC1) that reciprocally regulate the morning and evening loops. For example, the CCA1 and LHY in the morning loop activate the pseudo-response regulators (PRRs), including PRR5, PRR7, and PRR9, which in turn inhibit the expression of *CCA1* and *LHY* genes. On the other hand, the evening complex (EC) in the evening loop, which is a protein complex consisting of EARLY FLOWERING 3 (ELF3), EARLY FLOWERING 4 (ELF4), and LUX ARRHYTHMO (LUX) proteins, inhibits the expression of *PRR7* and *PRR9* (Figure 1). It is important to note that the EC components are themselves rhythmic, commonly through repression by the products of the CCA1 and LHY [6].

With the declaration of 2016 as the International Year of Pulses, the importance of pulse utilization and development in supporting sustainable food systems has been widely recognized [7]. Pulses, the edible seeds of legumes, are generally considered a good and affordable source of protein and fibers [8,9]. Moreover, legumes are prominent nitrogen fixers that can help improve soil quality and fertility [10]. In recent years, some underutilized legumes, such as winged bean (*Psophocarpus tetragonolobus*) and lablab (*Lablab purpureus*), have been promoted as climate-resilient crops, playing a critical role in achieving a suite of the Sustainable Development Goals (SDGs) that focus on tackling global food insecurity and climate change [8]. The targeted goals include, among others, Zero Hunger (Goal 2), Good Health and Well-being (Goal 3), and Climate Action (Goal 13). Although there is an increasing interest in legume biology, numerous knowledge gaps have yet to be filled for these crops, including the molecular basis and physiological importance of their circadian rhythms [7]. This review provides insights into the molecular components of circadian rhythms in the legume family *Leguminosae* (or *Fabaceae*), highlighting key findings in their clock research as well as the gaps in the existing literature. Based on published work on several well-studied leguminous species such as soybean (*Glycine max*), and common pea (*Pisum sativum*), we conclude that a more comprehensive understanding of the biological clock in legumes, especially how tissue-specific clocks are coordinated and how these connect to regulate development and physiology, is essential for genotype selection and improving the physiological adaptations of leguminous crops to climate change in the 21st century.

## 2. Defining the Important Components of Clock Research

**Figure 2:**
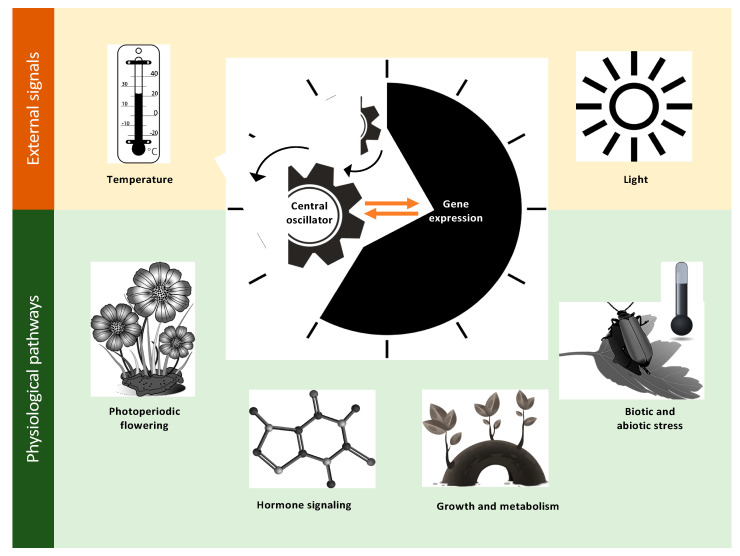
Light and temperature as examples of environmental signals integrated by the central oscillator of a plant to coordinate major physiological processes, including photoperiodic flowering, growth and metabolism, hormone signaling, and responses to biotic and abiotic stress in the natural environment.

The circadian system is a highly intricate network, with extensive crosstalk among output pathways that can be influenced by external conditions. External (or environmental) signals, such as light and temperature, are integrated by the central oscillator that coordinates various physiological processes [3,11]. A proper regular clock function is essential for the coordination of multiple physiological pathways, which include flowering time, growth and metabolism, hormone signaling, and responses to biotic and abiotic stresses (Figure 2). The orchestration of these complex interconnections yields a robust network that is paramount to the coordination of plant physiology in natural environments [5,12]. In Arabidopsis, the circadian clock system consists of a central oscillator that generates the endogenous circadian rhythms with input and output pathways that integrate environmental cues to the oscillator function and control various physiological processes, respectively. While light is considered the main signal that alters the plant circadian clock [13], many studies have revealed that the feedback regulation of the Arabidopsis clock is also affected by a wide spectrum of stress signals [1,14,15].

### 2.1. Photoperiodic Flowering

Flowering time, the period at which a plant produces the first floral bud, is a key factor associated with adaptation and yield responses of a particular species to various environments, locations, and agricultural practices [16]. Although morphological and flowering time variations have been documented in many crop legumes, most molecular studies of flowering time control have focused on the short-day (SD) legume soybean and the long-day (LD) legume common pea [17]. These studies, along with genetic analyses, such as reverse genetics in the model legumes barrel medic (*Medicago truncatula*) and birds-foot trefoil (*Lotus japonicus*), have laid a foundation for the exploration and characterization of flowering genes in a range of other legumes [18,19,20]. Within the papilionoid legumes, the galegoid and phaseoloid are two main sister clades that host major cool-season (such as common pea and chickpea) and warm-season (soybean and common bean) crop legumes, respectively [21]. In most cases, species within the galegoid clade are LD plants from temperate regions while those in the phaseoloid clade are SD plants from lower latitudes. The galegoid legumes were reported to have only a single ortholog for *CCA1* and *LHY* [22,23], while other clock genes, including the *TOC1*, *GI*, and *ELF3* are present as duplicate copies in some species within the two legume groups. Interestingly, the CONSTANS protein, which promotes flowering in LD plants such as Arabidopsis, is represented by only one co-ortholog in the LD legumes and two in the SD legumes [24].

Both genes and environmental factors affect the vegetative-to-reproductive transition in some leguminous species. In a broad sense, flowering time plays a fundamental role in deciding when and how a plant can allocate resources, participating in a complex network of interactions with other developmental processes [14]. The *FLOWERING LOCUS T/TERMINAL FLOWER 1* (*FT*/*TFL1*) gene family, which integrates environmental signaling for floral induction in Arabidopsis, has expanded in legumes and was reported to control the fate of meristems during flowering. Both galegoid and phaseoloid legumes have multiple *TFL1* genes and generally have three distinct subclades of *FT* gene (*FTa*, *FTb*, and *FTc*) [25]. Additionally, the MADS-box gene family, which regulates flower organ identity, has been characterized in certain legumes, such as barrel medic and soybean, where additional *SHORT VEGETATIVE PHASE* (*SVP*) and *SUPPRESSOR OF OVEREXPRESSION OF CONSTANS 1* (*SOC1*) genes were found [17,23].

### 2.2. Growth and Metabolism

Plants with circadian rhythms that synchronize with their environment are reported to gain growth or metabolic advantages over those that are not synchronized [12,26]. The present understanding of how the environment affects plant circadian rhythms came mainly from studies with Arabidopsis where regulation is mainly composed of three transcription feedback loops—the central, morning, and evening loops [12,27]. This functional configuration allows the clock to work in a rhythmic manner within a cycle of approximately 24 h. Dysfunctional clocks can decrease visible leaf area and net photosynthesis, leading to distorted development and reduced biomass and grain yield [28,29]. Studies on a handful of crop species, including the major legume soybean, suggested that shifting the circadian phase of key biological processes, such as photosynthesis, could impact the growth and development of the crops. For example, Pan et al. [29] reported the significant relationship between circadian rhythms and *Lhcb* (*CAB2* and *CAB1*) transcripts associated with chlorophyll synthesis and photosynthesis. These genes encode light-harvesting chlorophyll-binding proteins that form up to 50% of the thylakoid membrane protein and have cis-elements associated both with light-induced transcription and circadian control [29].

### 2.3. Hormone Signaling

Circadian and dial regulation of the levels of phytohormones is widespread in plants, likely involving a complex network of hormone signaling pathways. Circadian oscillations in the major growth-related phytohormones, including ethylene (ET), auxin/indole-3-acetic acid proteins (Aux/IAAs), cytokinins (CKs), gibberellins (GAs), and brassinosteroids (BRs), have been studied in multiple species that demonstrated species- and/or tissue-specific variations [30,31,32,33,34,35]. It is worth noting that defense-related hormones such as jasmonic acid (JA) and salicylic acid (SA) also undergo circadian oscillations. For instance, JA and SA levels have been found to be clock regulated in Arabidopsis, with peak accumulation in the middle of the day and night, respectively [36].

There are several ways in which the plant circadian system can interact with hormone metabolic pathways. The expression of specific genes that encode hormone biosynthesis enzymes in plants has been reported to be clock regulated. For example, many GA biosynthesis genes demonstrated regular daily rhythms in Arabidopsis and common pea [37,38], whereby the plant responsiveness to GA is controlled by the clock [39]. It was reported that the plant circadian clock regulates more than 50% of the genes that encode major enzymes of the methylerythritol phosphate (MEP) and mevalonate (MVA) pathways, which involve the synthesis of isoprenoid, the precursor of certain phytohormones (such as GAs, BRs, and CKs). In Arabidopsis, key genes in the MEP pathway were found to be controlled by the central clock proteins CCA1 and LHY [40]. IAA, the most studied auxin, is a key regulator of growth and development in plants that is derived mainly from tryptophan via the tryptophan aminotransferase/flavin monooxygenase (YUCCA) pathway. Rawat et al. [41] reported that the clock regulates auxin levels through a mechanism involving the circadian-regulated MYB-like transcription factor RVE1, which directly promotes the expression of the auxin biosynthesis gene *YUCCA8*, leading to increased auxin production during the day. In Arabidopsis, the daily rhythms in ABA, BR, IAA, and GA signaling are complex and modulated at many steps [42]. Although emissions of major phytohormones have long been recognized as clock-controlled, the complex mechanisms underlying these regulations in legumes remain elusive.

### 2.4. Biotic and Abiotic Stress

Apart from light and temperature, biotic and abiotic stresses activate plant response pathways that can alter the plant circadian clock. Little is known about the impacts of these stresses on the circadian clock of crop plant species, although major clock genes that are associated with some agronomic traits have been reported. The limited understanding of how different stresses can influence crop circadian clocks is considered one of the key knowledge gaps that impedes the identification of specific circadian traits for further germplasm improvement in breeding programs. Among legumes, abiotic stresses such as heat, drought, and iron deficiency have been reported to change the timing of the expression of core clock genes in soybean, with different stresses causing different physiological effects [43]. However, the interconnection between the changes in clock gene expressions and physiological changes remains elusive [44]. The study conducted by Li et al. [43] revealed that different soybean varieties with different iron uptake efficiencies may involve phase modulation as a mechanism to alleviate iron deficiency symptoms, demonstrating that it is important to dissect crop circadian clocks. Deciphering the complex interaction network between crop circadian clock and stress signals can help enhance abiotic stress tolerance of the crop. For example, ectopic expression of the Arabidopsis light-signaling B-box domain gene *AtBBX32* in soybean was found to increase the grain yield of multiple transgenic events in field trials [45].

## 3. Legume Clock Research at a Glance

**Figure 3:**
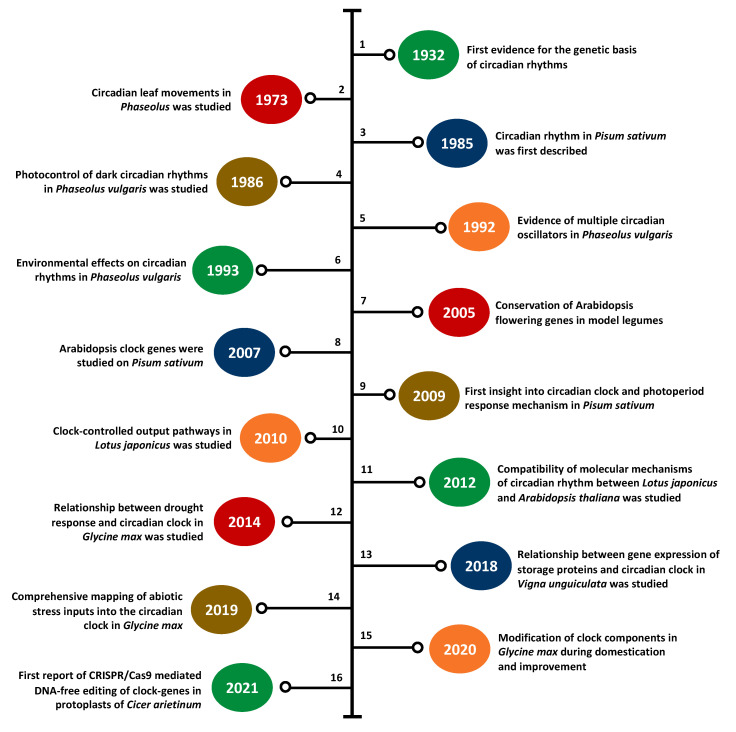
Timeline of major findings in clock research in legumes since the 1930s. 1—Bünning (1932) [71], 2—Bünning (1973) [72], 3—Kloppstsech (1985) [73], 4—Holmes and Klein (1986) [74], 5—Hennessey and Field (1992) [75], 6—Hennessey et al., (1993) [76], 7—Hecht et al., (2005) [17], 8—Hecht et al., (2007) [22], 9—Weller et al., (2009) [77], 10—Ono et al., (2010) [78], 11—Ueoka-Nakanishi et al., (2012) [50], 12—Marcolino-Gomes et al., (2014) [79], 13—Weiss et al., (2018) [70], 14—Li et el. (2019) [43], 15—Li et al. (2020) [80], 16—Badhan et al. (2021) [81].

While the first scientific knowledge of the plant circadian clock was reported nearly 300 years ago, the role of the molecular clock in growth and development is not fully understood and many clock-related components remain to be discovered [1,46]. Recent advances in genetics and genomics have paved the way for a better understanding of clock genes and circadian rhythmicity in crop legumes, particularly for popular species such as soybean and common pea. Over the past two decades, there has been a noticeable increase in the number of leguminous species used to elucidate the role of the clock-related components, including both major and minor (or underutilized) species (Table 1) [17,22,47,48,49,50,51,52,53,54,55,56,57,58,59,60,61,62,63,64,65,66,67,68,69,70]. Figure 3 presents the timeline of important clock research in legumes since the 1930s [17,22,43,50,70,71,72,73,74,75,76,77,78,79,80,81]. This section discusses the key findings in the clock research of these legumes and the critical gaps in the existing literature.

### 3.1. Clock Research in Model Legumes

#### 3.1.1. Barrel Medic

Barrel medic (*Medicago truncatula*) is a tiny annual legume that is used widely in legume genomics research. Native to the Mediterranean region, this forage legume has been used to study many aspects of plant biology, especially since the first release of a genome sequence in 2011 [82]. Many effective transformation methods, such as *Agrobacterium tumefaciens*-mediated and *A. rhizogenes*-mediated hairy root transformation, have been developed for functional genomic studies in this model legume [83]. Moreover, the ability of barrel medic to be efficiently nodulated by rhizobium has contributed to a better understanding of plant–microbe interactions, particularly the symbiotic relationship between various species of legumes and nitrogen-fixing bacteria [84,85].

Recently, Ma et al. [86] reported a comprehensive analysis of 36 circadian-related genes, namely those containing the CCT domain. The *CCT* (*CONSTANTS-CONSTANS LIKE-TIMING OF CAB 1*) genes have been previously implicated in flowering time regulation and biomass accumulation [87]. Gene clustering analysis for barrel medic associated the *CCT* genes with flowering time regulation, abiotic stress response, and regulation of growth and development [86]. A recent study revealed that the *LHY* (*LATE ELONGATED HYPOCOTYLS*) gene also plays an essential role in the regulation of the endogenous biological clock in barrel medic [48]. Functional loss of *MtLHY* can severely impact the transcription of several key circadian genes, and its over-expression may result in delayed flowering and hypocotyl elongation. The study also reported reduced nitrogen fixation with altered biological clocks in *Mtlhy* mutants, causing abnormal nyctinastic leaf movement and biomass reduction. The amenability of barrel medic to be genetically transformed by various methods has made it well-accepted as a model legume species that likely will continue to attract interest from crop researchers across different biological disciplines.

#### 3.1.2. Birds-Foot Trefoil

Similar to barrel medic, birds-foot trefoil (*Lotus japonicus*) is physically small and has a short generation time [54], allowing easy cultivation with transformation systems and multiple ecotypes and mutant resources available. Duangkhet et al. [88] studied the induction of *Ljmybr*, an MYB-related gene, with a possible role in nitrogen fixation regulation. The study identified *Ljmybr* as a possible *CCA1*-related gene sharing a conserved SHAQKY domain. *MYB* transcription factors are known to be associated with plant development, response to environmental signals, and hormone regulation [89]. MYB proteins of the CCA1-like subgroup, in particular, have been linked to biological clock regulation in soybean, maize (*Zea mays*), and Arabidopsis [90]. A comprehensive study on clock-associated F-box proteins, which are one of the main components of the S-phase-kinase-associated protein 1 (SKP1), Cullin-1, and F-box protein (SCF)-complex, conducted on three model species—birds-foot trefoil, barrel medic, and Arabidopsis—indicated that F-box proteins could be conserved among these model plants and play a crucial role in their growth and development [91]. In particular, the F-box proteins were associated with embryogenesis and nodulation. Further knockdown experiments highlighted the possible role of F-box proteins in cell cycle control, and that these proteins could be conserved among all three model plants [91]. A recent genome-wide association study on birds-foot trefoil found that clock-regulated adaptive flowering time traits and phenotype association signals for overwintering were direct targets of selection during colonization of Japan, indicating that these traits are critical for legume adaptation to cold climates [92].

#### 3.1.3. Soybean

Soybean (*Glycine max*), a globally traded legume, serves as a prime source of protein and vegetable oils for human consumption. Deemed the “king of beans”, this major legume is also widely used for animal feed and biofuel production. Recent years have seen rapid improvement in the quantity and quality of soybean seeds due mainly to the advances in genetic and metabolic engineering [93]. Although the first soybean transformation was reported in the late 1980s, the stable transformation of soybeans remains one of the major challenges because of its low transformation and regeneration efficiency [87,93,94]. Nonetheless, genetic engineering has been widely used in soybean, especially for the improvement of its nutritional value where significant efforts have been devoted to securing the global need for biofortified food [80].

Among the major evening complex genes such as *ELF3*, *ELF4*, and *LUX* found in plants, the *GmELF4* gene of soybean has been functionally characterized [72]. Similar to the function of *AtELF4* in Arabidopsis, *GmELF4* acts as a negative controller of flowering, delaying flowering via a range of unidentified molecular pathways. Other clock components that have been studied in soybean include *GmLCL1*, *GmTOC1*, *GmPRR9*, and *GmGI*, which revealed an arsenic stress response that can affect physiological responses, such as stomatal aperture, that naturally exhibit diurnal oscillations [44]. Arsenic, which is commonly found in soil as arsenate and arsenite, is an extremely toxic metalloid that affects soybean growth and productivity. However, further studies are required to fully understand the link between arsenic exposure and the circadian rhythm of soybean [44]. The circadian clock components in soybean are known to be modified as a result of domestication and improvement, and further studies are needed to exploit these modifications to improve soybean productivity [80].

#### 3.1.4. Common Pea

Another common crop legume is the common pea (*Pisum sativum*), which is also the model plant used by the father of genetics, Gregor Mendel, to study the laws of inheritance. Since the first description of circadian rhythm in common pea [73], a considerable body of research has been conducted concerning its various molecular pathways, ranging from temperature stress response [95,96] to the diurnal regulation of axillary budding [97]. Understanding the response to abiotic stressors, particularly heat and cold stresses, forms a fundamental portion of pea molecular research. Being a cool season legume, it is generally more sensitive to heat stress as compared to its warm season counterparts such as pigeon pea and mungbean. The study conducted by García-García et al. [97] unraveled the oxidative response of both common pea and bean to sustained irrigation deficit and concluded that the value of these legumes as dietary sources of bioactive compounds depends on species, variety, and also growing conditions.

The first research into the circadian clock and photoperiod response mechanism in common pea was reported by Weller et al. [77], highlighting progress in gene and mutant isolation, candidate gene assessment, expression analyses, and physiological studies. The study identified several loci as orthologues of *AtGIGANTEA* (or *AtGI*) and *AtELF4*, including *LATE BLOOMER1* (*LATE1*) and *DIE NEUTRALIS* (*DNE*). According to Liew et al. [62], the *STERILE NODES* (*SN*) locus in common pea was among the first photoperiod response genes to be studied, demonstrating the genetic control of flowering-time regulation through long-distance signaling. The study reported that *SN* formed a complex network with two other circadian clock genes, *DNE* and *HIGH RESPONSE TO PHOTOPERIOD* (*HR*), where *HR* regulates the expression of *SN* while *SN* influences the role of *HR* and *DNE* in controlling flowering. The common pea axillary bud transcriptome analysis demonstrated rapid changes in the temporal expression of diurnally regulated genes within the short 170-min time frame, suggesting that future gene expression studies in this crop should take into account the possibility of fast diurnal changes in gene expression [98].

### 3.2. Clock Research in Underutilized Legumes

During the past decade, it has been observed that unlocking the potential of underutilized legumes, such as winged bean and lentil, is equally important as the improvement of major legumes such as soybean and common pea [7,8]. These leguminous species have recently been promoted as the protein alternatives to soybean and meat, being part of the solution to future food and nutrition insecurity amidst climate change [8]. However, the current state of knowledge of circadian clocks in leguminous species stems primarily from the research on the model and/or economically important legume species as discussed in Section 3.1 and Section 3.2. Clock research in underutilized legumes is scarce in literature. One notable study is the identification of an *ELF3* homolog in lentil, along with common pea (*HR* locus), as the genetic factor underlying flowering time variation [23]. More research to understand the molecular mechanisms of circadian rhythmicity in potential underutilized crops is crucial to help ensure the sustainability for future food or protein. All things considered, optimizing circadian function can offer opportunities to enhance the productivity of potential legumes and crops in general, including those grown over broad latitudinal ranges [3].

## 4. Concluding Remarks and Perspective

The past two decades have seen enormous progress in understanding the molecular features and architecture of circadian oscillators in plants. Nonetheless, knowledge of the biological and biochemical functions of some core clock components is still lacking, especially in non-model plants. The increasing evidence of novel molecular mechanisms that play a role in circadian rhythmicity calls for more comprehensive research to understand and validate these mechanisms. Recent studies have revealed the presence of tissue-specific clocks in plants that are regulated in a reciprocal fashion, demonstrating more complex and distinct rhythmic properties in the network that had previously been overlooked. More focus should be given to elucidate the molecular mechanisms of these tissue-specific clocks and determine how they communicate with each other to enhance the growth and fitness of plants. Specifically, crop fitness has long been artificially selected through breeding and domestication, and the circadian clock system has been identified as a key to improved fitness through selections. Despite significant progress in Arabidopsis and a few other model crop species, the present knowledge of the crop circadian clock is generally still limited. It is crucial to gain a more precise understanding of the relevant connections between the physiological pathways and the clock’s overall effects on plant development.

Early clock research in plants has focused primarily on Arabidopsis—the model plant that has defined many circadian clock components and functions in plants, which suggests that the available knowledge of the overall circadian system may somehow be distorted [99]. For crop improvement research that is based on circadian regulators, it is important to obtain a more comprehensive description of time-course resolution for transcript abundance levels to verify if circadian patterns observed in the model plant Arabidopsis are similar or maintained in other plant species, especially those with different ploidy levels. During the past decade, the transition of clock research from Arabidopsis to newer model systems for monocot (such as rice) and dicot (such as soybean and common pea) crops has targeted mainly at exploring the potential functions of clock genes associated with growth and physiological processes, which are crucial to improve crop yield in a rapidly changing world. The rhythmic changes in transcript abundance that affect physiological processes due to changes in temperature and light have been revealed by a number of transcriptome studies. One of the challenges to identify new or overlapping functions among paralogs is the heterogeneity in transcriptome datasets, which are often generated from various tissue types at specific times under various environmental conditions. More time-course transcriptomics studies should be carried out to identify regulatory elements, including the roles of epigenetic regulators that may contribute to circadian network interactions and identification of the changes or the evolution of new forms of transcriptional control of different plant species.

Circadian clocks play a critical role in plant development and physiology and thus are key components for inclusion in legume crop selection and improvement strategies. Although several core clock genes have been studied in some leguminous species [53], functional annotations of most of these genes have yet to be performed. Omics approaches including genome-wide gene expression analysis can provide supporting data for a more in-depth understanding of biological clock mechanisms in legumes, especially how tissue-specific clocks are coordinated to regulate their development, physiology and responses to environmental cues other than light. This information is essential for genotype selection and to improve the physiological adaptations of current and future leguminous crops to climate change in the 21st century.

## Figures and Tables

**Table 1:** Specific circadian rhythm genes studied in model and/or major legumes.

Species	Gene	Arabidopsis Homologue	Function(s)	Reference(s)
Barrel medic (*Medicago truncatula*)	*MtGI*	*GI*	Circadian clock component, photoperiod response	[47]
	*MtLHY*	*LHY*	Regulation of circadian rhythm in nodules and nyctinastic leaf movement	[48]
Birds-foot trefoil (*Lotus japonicus*)	*LjaPRR5*	*PRR5*	Component of the circadian rhythm	[49]
	*LjaPRR7*	*PRR7*		
	*LjaPRR9*	*PRR9*		
	*LjaLUX*	*LUX*		
	*LjaTOC1*	*TOC1*	Central component of the circadian rhythm	
	*LjaLHY*	*LHY*		
	*LjCCA1*	*CCA1*	Central component of the circadian rhythm	[50]
	*LjGI*	*GI*	Possible regulation of flowering time	[51]
Soybean (*Glycine max*)	*GmTOC1*	*TOC1*	Central component of the soybean circadian clock (expressed as an evening gene)	[52]
	*GmELF4*	*ELF4*	Circadian clock function.	[53]
	*GmGIa*	*GI*	Photoperiod response, flowering time regulation	[54]
	*GmLCL1*	*LHY/CCA1*	Central component of the soybean circadian clock (expressed as a morning gene)	[52]
	*GmLCL2*			
	*GmLHY*	*LHY*	Regulate plant height	[55]
	*GmLUXa*	*LUX*	Control flowering time	[56]
	*GmLUXb*			
	*GmLUXc*			
	*GmZTL3*	*ZTL*	Control of flowering time (inhibitor of flowering induction) and photoreceptor.	[57]
	*GmPRR37*	*PRR3 & 7*	Control of soybean photoperiodic flowering	[58]
Common pea (*Pisum sativum*)	*PsTOC1*	*TOC1*	Circadian clock component	[17,59]
	*DNE*	*ELF4*	Circadian clock component, flowering time regulation	[60,61]
	*HR*	*ELF3*	Circadian clock component, flowering time regulation, light response	[61]
	*LATE1*	*GI*	Photoperiod response	[22,60]
	*MYB1/LHY*	*CCA1/LHY*	Circadian clock component	[60]
	*SN*	*LUX*	Circadian clock component	[62]
	*PsPRR37*	*PRR*	Component of phospho-relay signal transduction system	[59]
	*PsPRR59*			
Chickpea (*Cicer arietinum*)	*Efl1*	*ELF3*	Flowering regulation (light input to the circadian clock)	[63]
	*GI*	*GI*	Flowering time regulation	[64]
Common bean (*Phaseolus vulgaris*)	*PvLHY*	*LHY*	Circadian mechanism regulation	[65,66]
	*PvTOC1*	*TOC1*	Mediating light responsiveness to circadian clock mechanism	[67,68]
	*PvELF4*	*ELF4*	Evening-expressed gene	[67]
	*PvGI*	*GI*	Circadian clock component	[69]
	*PvZTL*	*ZTL*		
Pigeon pea (*Cajanus cajan*)	*CcGI*	*GI*	Determinacy and flower patterning	[69]
Cowpea (*Vigna unguiculata*)	*VunTOC1*	*TOC1*	Circadian clock function in seed filling and leaves	[70]
	*VunLHY*	*LHY*		
	*VunELF3*	*ELF3*		
	*VunGI*	*GI*		
Lentil (*Lens culinaris*)	*HR*	*ELF3*	Flowering time regulation	[61]
White lupin (*Lupinis albus*)	*GI*	*GI*	Flowering regulation; anthracnose resistance	[64]
